# Genetic variations in miR‐125 family and the survival of non‐small cell lung cancer in Chinese population

**DOI:** 10.1002/cam4.2073

**Published:** 2019-03-07

**Authors:** Shuangshuang Wu, Wei Shen, Lu Yang, Meng Zhu, Mingjiong Zhang, Feng Zong, Liguo Geng, Yuzhuo Wang, Tongtong Huang, Yun Pan, Songyu Cao, Juncheng Dai, HongXia Ma, Jianqing Wu

**Affiliations:** ^1^ Jiangsu Provincial Key Laboratory of Geriatrics, Department of Geriatrics the First Affiliated Hospital with Nanjing Medical University Nanjing China; ^2^ Department of Epidemiology, School of Public Health Nanjing Medical University Nanjing China; ^3^ Department of Laboratory Medicine The First Affiliated Hospital with Nanjing Medical University Nanjing China; ^4^ Jiangsu Key Lab of Cancer Biomarkers, Prevention and Treatment, Collaborative Innovation Center For Cancer Personalized Medicine, School of Public Health Nanjing Medical University Nanjing China; ^5^ Department of Information The Affiliated Drum Tower Hospital of Nanjing University Medical School Nanjing China; ^6^ Editorial Department of Journal of Clinical Dermatology the First Affiliated Hospital with Nanjing Medical University Nanjing China

**Keywords:** miR‐125, miRNAs, non‐small cell lung cancer, single nucleotide polymorphisms, survival

## Abstract

To investigate the associations between the functional single nucleotide polymorphisms (SNPs) in the miR‐125 family and the survival of non‐small cell lung cancer (NSCLC) patients, we systematically selected six functional SNPs located in three pre‐miRNAs (miR‐125a, miR‐125b‐1, miR‐125b‐2). Cox proportional hazard regression analyses were conducted to estimate the crude and adjusted hazard ratios (HRs) and their 95% confidence intervals (CIs). Reporter gene luciferase assay was performed to examine the relationship between the SNPs and transcriptive activity of the miRNAs. The expression of miRNAs in different cells was detected using quantitative real‐time PCR assay. We found that rs2241490 (upstream of miR‐125b‐1, G > A, adjusted HR = 1.24, 95%CI = 1.05‐1.48, *P = *0.014, in dominant model; adjusted HR = 1.18, 95%CI = 1.03‐1.35, *P* = 0.014, in additive model), rs512932 (upstream of miR‐125b‐1, A > G, dominant model: adjusted HR = 1.25, 95%CI = 1.05‐1.48, *P = *0.013) and rs8111742 (upstream of miR‐125a, G > A, dominant model: adjusted HR = 0.84, 95%CI = 0.71‐1.00, *P = *0.047) were associated with the prognosis of 1001 Chinese NSCLC patients. The combined analysis of the three SNPs related the number of risk alleles (rs2241490‐A, rs512932‐G and rs8111742‐G) to death risk of NSCLC in a locus‐dosage mode (*P* for trend <0.001). Furthermore, luciferase reporter gene assay showed significantly higher levels of luciferase activity with rs512932 variant G than that with A allele in 293T, SPC‐A1 and A549 cell lines. Besides, miR‐125b was highly expressed in lung cancer cells than the normal lung cell. Our study indicated that genetic variations in miR‐125 family were implicated in the survival of NSCLC patients. Larger population‐based and functional studies are needed to verify these findings.

## 1 INTRODUCTION

Lung cancer is among the leading cause of cancer‐related death globally, with the 5‐year survival rate generally lower than 20% around the world.^1^ The proportion of non‐small cell lung cancer (NSCLC) is 80% in all the lung cancer cases. The tumor‐node‐metastasis staging system has traditionally and extensively been used to evaluate the prognosis and determine the most rational management of cancer patients. However, there are remarkable differences in recurrence and survival within the same staging group, indicating the inadequacy of the staging system to account for this heterogeneity. Therefore, it is necessary to develop specific prognostic biomarkers to help improve cancer detection, treatment and prognosis.^2^

MicroRNAs (miRNAs) belong to endogenous small noncoding and single stranded RNAs with 19 ~ 25 nucleotides. They predominantly bind to the 3’ untranslated region (UTR) or 5’UTR of the targeted mRNAs, thus regulating the abundance of mRNAs and the expression of the corresponding proteins.^3^ A single miRNA can bind to hundreds of mRNA targets, thereby implicating in crucial biological processes.^4^, ^5^, ^6^ Studies have found significantly different miRNA profiles in tumors, indicating that miRNAs play substantial roles in the development, progression and survival of cancers,^7^ including lung cancer.^8^, ^9^, ^10^ As the human homolog of the miRNA lin‐4, which was the first discovered miRNA in C. elegans development,^11^ miR‐125 family consists of hsa‐miR‐125a, hsa‐miR‐125b‐1 and hsa‐miR‐125b‐2. Members of the family play crucial roles in tumorigenesis and progression, mainly regulating tumor cell growth in immunity, proliferation, apoptosis, and invasion or metastasis.^12^, ^13^, ^14^, ^15^ Expression of different members in miR‐125 family has controversial properties in lung cancer.^15^, ^16^

Germline sequence abnormalities such as single nucleotide polymorphisms (SNPs) identified in miRNA genes can affect the transcription of primary transcripts (pri‐miRNAs), precursor RNAs (pre‐miRNAs) processing and maturation, or miRNA‐mRNA interactions.^17^, ^18^, ^19^ For instance, the miR‐30c‐1 rs928508 G allele was associated with significantly decreased expression levels of pre‐ and mature‐miR‐30c‐1 via modulating the pri‐miRNA processing, subsequently resulted in better NSCLC survival.^20^ As each miRNA has various targets, the inherited and miRNA expression‐related minor variations might have significant influences on the expression of various protein‐coding genes which are implicated in malignant transformation.

According to the indicative important role of miR‐125 family and SNPs in cancer development, we hypothesized that the SNPs in the region of miR‐125 family (hsa‐miR‐125a, hsa‐miR‐125b‐1 and hsa‐miR‐125b‐2) genes may have effects on clinical outcome in NSCLC patients, possibly through influencing the expression of miR‐125a/b. We selected six potentially functional SNPs located in and 10kb upstream regions of the three pre‐miRNAs and tested the association between these SNPs and survival of Chinese NSCLC patients.

## 2 MATERIALS AND METHODS

### 2.1 Study population

We recruited newly diagnosed patients who were confirmed as NSCLC by histopathologic or cytologic examinations and without other cancer history, from the Cancer Hospital of Jiangsu Province, and the First Affiliated Hospital of Nanjing Medical University (NMU) (Nanjing, China). The diagnosis was reviewed by at least two pathologists. A standard questionnaire was administered through face‐to‐face interviews to obtain demographic data and lifestyle factors, including sex, age, and cigarette smoking. Those who smoked at least one cigarette per day and over one year throughout their lifetime were defined as smokers; otherwise, they were considered as nonsmokers. Each patient donated 5 mL of fasting venous blood sample and was followed up every 3 months to collect the information of treatment and progression. From July 2003 to August 2013, a total of 1341 NSCLC patients have been prospectively recruited, among which 1001 cases (74.6%) had complete demographic and subsequent follow‐up information and sufficient DNA specimens, with a median survival time (MST) of 26.0 months.^21^ The median follow‐up time of the patient cohort was 18.8 months for all patients. Our study was authorized by the Institutional Review Board in NMU. All the participants have signed informed consent before participating in the study.

### 2.2 SNP selection and genotyping

We focused on potentially functional SNPs of the selected pre‐miRNAs. SNPs located in or within 10kb upstream of hsa‐miR‐125a, hsa‐miR‐125b‐1 and hsa‐miR‐125b‐2, were extracted from the HapMap database (phase II +III Feb 09, on NCBI B36 assembly, dbSNP b126). SNPs meeting the following criteria were included in our study: (a) having a minor allele frequency (MAF) ≥ 0.05 in population of Han Chinese in Beijing (CHB); (b) satisfying Hardy‐Weinberg equilibrium (HWE) (*P* ≥ 0.05); (c) with genotyping rate ≥90%. SNPs were further annotated using SNPinfo Web Server (http://snpinfo.niehs.nih.gov/). When multiple SNPs were in strong linkage disequilibrium (*r* ^2^ ≥ 0.8), we kept only one SNP. As a result, six SNPs were selected. Genomic DNA was isolated from leukocytes of venous blood via proteinase K digestion and then extracted by phenol‐chloroform. The genotyping was implemented on Illumina Infinium® BeadChip (Illumina Inc). The information of assay conditions and the primers and probes is available if requested. Quality control strategies (ie, one blank well and three repeated samples) were strictly followed, as described in our previous studies.^21^ Finally, the six SNPs were all successfully genotyped with genotyping rates above 95%.

### 2.3 Cell culture

The human lung cancer cells (A549, SPC‐A1, H460, and PC9), 16 human bronchial epithelial cells (16HBE), and human embryonic kidney cells (293T) were purchased from the Shanghai Institute of Biochemistry and Cell Biology, Chinese Academy of Sciences (Shanghai, China). The cell lines were cultured in DMEM medium with 10% heat‐inactivated fetal bovine serum (Gibco, USA) and 100 ug/mL streptomycin (Gibco, USA) at a 37℃‐incubator supplemented with 5% CO_2_. We have authenticated the cells and did not find contamination from mycoplasma as well as cell line cross‐contamination.

### 2.4 Reporter gene luciferase assay

The 5'‐flanking region sequence of pre‐miR‐125b‐1 gene was obtained from the Homo sapiens chromosome 11 (NC_000011.10) after a blast search. The fragments from −1000 to the transcription start site (TSS) of the pre‐miR‐125b‐1(including rs2241490) and from −3590 to −2590 (including rs512932) sequences were separately synthesized and constructed into pGL3‐enhancer vector (Promega, Madison, WI, USA) by Generay Company (Shanghai, China). We validated all the plasmids by DNA sequencing.

For transfection, cells were seeded into 24‐well culture plates and transfected via lipofectamine‐2000 transfection reagent with 0.5 μg constructed luciferase reporter gene plasmids mentioned above. pRL‐SV40 (as internal control) was transiently co‐transfected into cells for correcting transfection efficiency. Twenty‐four hours after transfection, all cells were washed with PBS and lysed with 1 × passive lysis buffer. Luciferase activity was determined by the Dual‐Luciferase Reporter Assay System (Promega, Madison, WI) following the manufacturer's protocol. Each cell line was used in 3 independent transfection experiments, and each experiment was performed in quadruplicate.

### 2.5 RNA isolation and quantitative real‐time PCR assay

Total RNA from four types of cells was extracted by using the miRNeasy Mini kit (Qiagen, Germany) and then was reversely transcribed to complementary DNA with the TaqMan miRNA RT Kit and stem‐loop RT primers (Applied Biosystems, USA). miRNA expression was detected based on the TaqMan PCR kit implemented in the ABI 7900 real‐time PCR System (Applied Biosystems, USA) and normalized using the threshold cycle (Ct) of U6. All reactions were performed in triplicate.

### 2.6 Statistical analyses

Deviation status of genotype distribution from the HWE for each SNP was examined by a goodness‐of‐fit χ^2^ test. Survival time was determined starting from the date of lung cancer diagnosis to that of death or last follow‐up. Kaplan–Meier method and log‐rank test were used to compare the survival time among subgroups categorized by patient characteristics, clinical features and genotypes. Univariate and multivariate Cox proportional hazard regression analyses were conducted to estimate the crude and adjusted hazard ratios (HRs) and their 95% confidence intervals (CIs). Adjusted covariates included age, sex, cigarette smoking, clinical stage, histology, lung cancer surgery, and chemotherapy or radiotherapy status. The heterogeneity among subgroups was examined with the χ^2^‐based Q‐test. One‐way ANOVA and Scheffe method were used to compare the levels of luciferase activity among three groups and between each two groups in the condition of homogeneity of variance, respectively. Comparison of the miRNA levels in lung cancer cell lines with 16HBE was conducted by using Student's *t*‐test. All analyses were two‐sided with 0.05 as significant level and used R software (Version 3.3.2; the R Foundation for Statistical Computing). STATA (version 12.0; Stata Corp., College Station, TX) was used to plot the survival curves of patients with different allele combinations. And histograms of relative luciferase activity and miR‐125b expression level were plotted using MS Office Excel 2013.

## 3 RESULTS

As shown in Table 1, 1001 patients were included in the final analysis, among which 545 deaths were observed during the period of follow‐up. The median age at diagnosis was 62 years and 69.4% (n = 695) were males. There were 657 (65.6%) adenocarcinomas and 344 (34.4%) squamous cell carcinomas. Cigarette smoking, advanced clinical stage, and chemotherapy or radiotherapy were significantly associated with shorter survival time (log‐rank *P* < 0.05, Table 1). In contrast, female and lung cancer surgical resection could significantly ameliorate the prognosis of NSCLC (log‐rank *P* < 0.05, Table 1).

**Table 1:** Basic characteristics and clinical features for the 1001 NSCLC patients

Variables	Patients	Deaths	MST(mo)	Log‐rank *P*	HR (95% CI)
	N = 1001(%)	N = 545			
Age				0.418	
≤62	513 (51.2)	268	27.4		1
>62	488 (48.8)	277	25.8		1.07(0.91‐1.27)
Gender				0.034	
Male	695 (69.4)	399	25.0		1
Female	306 (30.6)	146	32.9		0.81 (0.67‐0.99)
Smoking				0.027	
Never	401 (40.1)	197	30.0		1
Ever	600 (59.9)	348	23.9		1.22 (1.02‐1.45)
Surgical operation				<0.001	
No	325 (32.5)	247	14.6		1
Yes	676 (67.5)	298	44.4		0.33 (0.27‐0.39)
Clinical stage^a^				<0.001	
I/II	417 (41.7)	165	59.3		1
III/IV	564 (56.3)	370	18.9		2.71 (2.25‐3.27)
Histology				0.060	
Squamous cell	344 (34.4)	198	22.2		1
Adenocarcinoma	657 (65.6)	347	28.5		0.85 (0.71‐1.01)
Chemotherapy or radiotherapy^a^				0.026	
No	236 (23.6)	110	30.8		1
Yes	757 (75.6)	430	25.6		1.27 (1.03‐1.56)

CI, confidence intervals; HR, hazard ratio; MST, median survival time; NSCLC, non‐small cell lung cancer.

^a^ Variable which includes missing data.

Two SNPs (rs512932, A > G, which is 2989bp in the upstream of hsa‐miR‐125b‐1 and rs8111742, G > A, which is 1033bp in the upstream of has‐miR‐125a) showed significance in log‐rank test in the dominant models (rs512932: *P = *0.007; rs8111742: *P = *0.030) (Table 2). Furthermore, the multivariate Cox regression analyses showed three SNPs as significant independent prognostic markers for NSCLC survival. rs2241490 (G > A, 228bp in the upstream of hsa‐miR‐125b‐1) was associated with worse NSCLC survival (dominant model: adjusted HR = 1.24, 95%CI = 1.05‐1.48, *P* = 0.014; additive model: adjusted HR = 1.18, 95%CI = 1.03‐1.35, *P = *0.014). The mutated G allele of rs512932 was shown to contribute to worse survival (dominant model: adjusted HR = 1.25, 95%CI = 1.05‐1.48, *P* = 0.013). In contrast, rs8111742 (G > A) was associated with better NSCLC survival (dominant model: adjusted HR = 0.84, 95%CI = 0.71‐1.00, *P* = 0.047) (Table 3).

**Table 2:** Distributions of six SNPs in the NSCLC patients and associations with the survival

Pre‐miRNA	SNP^a^	Location^b^	Patients (WH/H/VH)	Deaths (WH/H/VH)	Genotyping Rate (%)	MAF in patients	Log‐rank *P*		
							Additive	Dominant	Recessive
							model	model	model
*hsa‐miR‐125a*	rs10405559 (G > A)	chr19: 52191120	838/157/6	455/86/4	100.00	0.080	0.919	0.960	0.694
	rs8111742 (G > A)	chr19: 52195474	545/379/77	307/194/44	100.00	0.270	0.067	0.030	0.877
*hsa‐miR‐125b‐1*	rs2081443 (A > C)	chr11: 121970746	465/435/100	246/250/48	99.90	0.320	0.344	0.495	0.311
	rs2241490 (G > A)	chr11: 121970780	491/426/84	259/241/45	100.00	0.300	0.473	0.235	0.938
	rs512932 (A > G)	chr11: 121973541	549/376/75	288/217/40	99.90	0.260	0.017	0.007	0.939
*hsa‐miR‐125b‐2*	rs7279730 (A > G)	chr21: 17961544	723/249/29	404/122/19	100.00	0.150	0.481	0.832	0.278

MAF, minor allele frequency; NSCLC, non‐small cell lung cancer; SNP, single nucleotide polymorphism; WH/H/VH, Wild Homozygotes, Heterozygotes, and Variant Homozygotes.

^a^ SNP name (Major>minor allele).

^b^ Database based on NCBI36/hg19.

**Table 3:** The associations between three positive SNPs in the miR‐125 family and survival of the NSCLC patients

Genotype	Patients	Deaths	MST	Crude HR	Adjusted HR	*P* ^a^
			(mo)	(95% CI)	(95% CI)^a^	
rs2241490 (*mir‐125b‐1*)						
GG	491	259	28.2	1	1	
GA	426	241	23.9	1.12 (0.94‐1.33)	1.23 (1.03‐1.47)	0.025
AA	84	45	26.0	1.07 (0.78‐1.46)	1.33 (0.96‐1.82)	0.085
GA/AA	510	286	24.9	1.11 (0.94‐1.31)	1.24(1.05‐1.48)	0.014
Additive model				1.07 (0.94‐1.21)	1.18 (1.03‐1.35)	0.014
rs512932(*mir‐125b‐1*)						
AA	549	288	28.5	1	1	
AG	376	217	22.3	1.29 (1.08‐1.54)	1.30 (1.08‐1.55)	0.005
GG	75	40	31.8	1.12 (0.81‐1.56)	1.02 (0.73‐1.44)	0.889
AG/GG	451	257	23.2	1.26 (1.07‐1.49)	1.25 (1.05‐1.48)	0.013
Additive model				1.15 (1.01‐1.31)	1.12 (0.98‐1.28)	0.085
rs8111742(*mir‐125a*)						
GG	545	307	24.0	1	1	
GA	379	194	32.4	0.81 (0.67‐0.97)	0.81 (0.67‐0.97)	0.022
AA	77	44	22.4	0.94 (0.68‐1.29)	1.02 (0.74‐1.40)	0.927
GA/AA	456	238	29.2	0.83 (0.70‐0.98)	0.84 (0.71‐1.00)	0.047
Additive model				0.89 (0.78‐1.02)	0.91 (0.79‐1.05)	0.192
Combined analysis^b^						
0‐1	156	74	39.4	1	1	
2	319	172	25.9	1.27 (0.97‐1.67)	1.26 (0.95‐1.67)	0.103
3	339	193	25.0	1.40 (1.07‐1.83)	1.42 (1.08‐1.88)	0.012
≥4	186	106	24.0	1.56 (1.16‐2.11)	1.65 (1.22‐2.24)	0.001
Trend *P*						6.60E‐04

CI, confidence intervals; HR, hazard ratio; MST, median survival time; NSCLC, non‐small cell lung cancer; SNP, single nucleotide polymorphism.

^a^ Adjusted by age, gender, smoking status, clinical stage, chemotherapy or radiotherapy status, surgery status, and histology.

^b^ The combined genotypes were addition of risk alleles carried (rs2241490‐A, rs512932‐G and rs8111742‐G).

**Figure 1:**
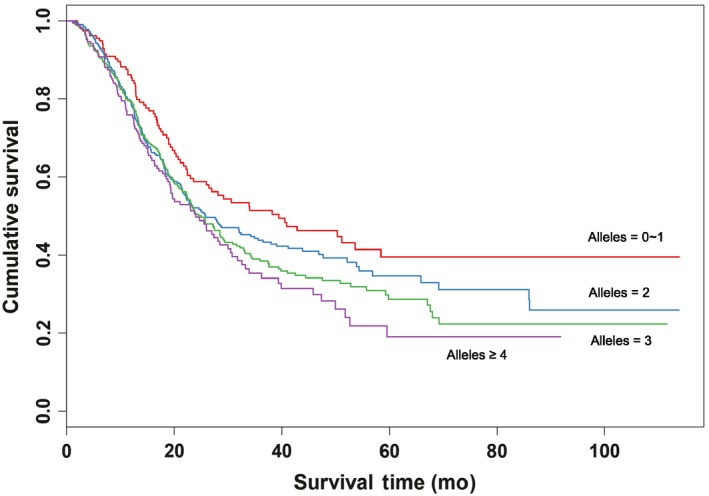
Kaplan–Meier plots of survival according to combined risk alleles of the three SNPs (rs2241490‐A, rs512932‐G and rs8111742‐G) in the Chinese NSCLC patients. SNP, single nucleotide polymorphism

Then we evaluated the combined effects of the three SNPs (rs2241490‐A, rs512932‐G and rs8111742‐G) on NSCLC survival. The results showed that the more risk alleles the patients carried, the shorter MST they would survive, suggesting a significant locus‐dosage effect between risk alleles and survival of NSCLC (*P *for trend <0.001). Compared to subjects with “0 ~ 1” risk allele (MST = 39.4 months), subjects who carried two or more risk alleles had shorter of MSTs (MST = 25.9, 25.0, 24.0 months for “2”, “3” and “≥4” risk alleles, respectively) and larger HR of 1.26 (95%CI = 0.95‐1.67), 1.42(1.08‐1.88), and 1.65(1.22‐2.24), respectively (Table 3, Figure 1).

To further characterize the relationship of the three SNPs with NSCLC survival, we conducted a stratified analysis according to age, sex, cigarette smoking, surgical operation, stage, histology, and chemotherapy or radiotherapy therapy in dominant model. As shown in Table 4, we found that rs2241490 had stronger risk effect on NSCLC survival in female patients and those with I/II clinical stage (*P = *0.036, *P = *0.024 for heterogeneity test, respectively). Similarly, rs512932 also had a stronger risk effect in patients with I/II stage (*P_Heterogeneity_* = 0.002). According to a further interaction analysis, statistically significant multiplicative interactions were found for rs512932 and rs2241490 with stage (both with *P_int_* < 0.001) (Table 5).

**Table 4:** Stratification analysis on association of the SNPs and the survival of NSCLC patients

Variables	Rs2241490 (G > A)		Rs512932 (A > G)		Rs8111742 (G > A)	
	HR (95% CI)^a^	*P* b	HR (95% CI)^a^	*P* b	HR (95% CI)^a^	*P* b
Age						
<62	1.25 (0.97‐1.59)	0.979	1.23 (0.96‐1.58)	0.864	0.93 (0.73‐1.19)	0.234
≥62	1.24 (0.97‐1.58)		1.27 (0.99‐1.62)		0.75 (0.59‐0.96)	
Gender						
Male	1.12 (0.92‐1.37)	0.036	1.17 (0.95‐1.43)	0.199	0.92 (0.75‐1.13)	0.076
Female	1.71 (1.22‐2.40)		1.51 (1.08‐2.11)		0.64 (0.45‐0.91)	
Smoking						
Never	1.53 (1.14‐2.05)	0.130	1.5 (1.12‐1.99)	0.120	0.78 (0.58‐1.04)	0.491
Ever	1.16 (0.93‐1.44)		1.12 (0.9‐1.4)		0.88 (0.71‐1.09)	
Surgical operation						
No	1.23 (0.94‐1.59)	0.913	1.28 (0.99‐1.67)	0.814	0.76 (0.58‐0.99)	0.341
Yes	1.25 (0.99‐1.58)		1.23 (0.97‐1.55)		0.9 (0.71‐1.14)	
Clinical stage						
I/II	1.68 (1.21‐2.34)	0.024	1.92 (1.40‐2.62)	0.002	0.87 (0.64‐1.19)	0.850
III/IV	1.07 (0.87‐1.32)		1.06 (0.86‐1.31)		0.84 (0.68‐1.03)	
Histology						
Squamous cell	1.13 (0.84‐1.51)	0.300	1.03 (0.77‐1.38)	0.112	0.99 (0.74‐1.32)	0.168
Adenocarcinoma	1.37 (1.10‐1.70)		1.39 (1.12‐1.72)		0.77 (0.62‐0.96)	
Chemotherapy or radiotherapy						
No	1.32(0.88‐1.96)	0.743	1.1 (0.74‐1.64)	0.551	0.78 (0.53‐1.15)	0.645
Yes	1.22(1.01‐1.48)		1.26 (1.04‐1.53)		0.86 (0.71‐1.05)	

CI, confidence intervals; HR, hazard ratio; MST, median survival time; NSCLC, non‐small cell lung cancer.

a Adjusted for age, gender, smoking status, clinical stage, chemotherapy or radiotherapy status, surgery status, histology except for the stratification factor. And the association studies were performed in dominant models.

b Heterogeneity test for differences between groups.

**Table 5:** The interaction analysis between the SNPs and clinical features

Variable	Clinical stage	Patients	Deaths	MST(mo)	Crude HR (95%CI)	Adjusted HR (95%CI)^a^	*P* a
Rs2241490 (G > A) genotypes							
GG	I/II	187	62	NA	1	1	
GG	III/IV	290	189	19.6	3.41 (2.56‐4.56)	2.58 (1.89‐3.52)	<0.001
GA/AA	I/II	230	103	49.9	1.53 (1.12‐2.10)	1.59 (1.15‐2.19)	0.005
GA/AA	III/IV	274	181	18.3	3.52 (2.63‐4.71)	2.78 (2.04‐3.79)	<0.001
*P* for multiplicative interaction							<0.001
Rs512932 (A > G) genotypes							
AA	I/II	234	78	85.9	1	1	
AA	III/IV	301	202	19.6	3.63 (2.79‐4.74)	2.70 (2.02‐3.60)	<0.001
AG/GG	I/II	183	87	39.4	1.87 (1.38‐2.54)	1.93 (1.41‐2.62)	<0.001
AG/GG	III/IV	262	168	18.1	3.59 (2.74‐4.71)	2.86 (2.15‐3.82)	<0.001
*P* for multiplicative interaction							<0.001

CI, confidence intervals; HR, hazard ratio; MST, median survival time; SNP, single nucleotide polymorphism.

a Adjusted for age, gender, smoking status, clinical stage, chemotherapy or radiotherapy status, surgery status, histology except for the interaction factor. And the association studies were performed in dominant models.

**Figure 2:**
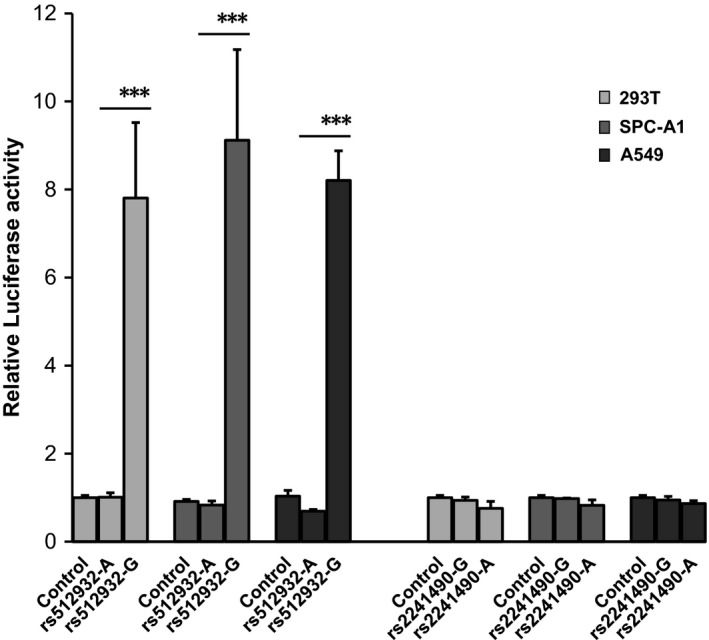
Different levels of luciferase activity of the region harboring rs512932 and rs2241490 in 293T, SPC‐A1, and A549 cell lines. All constructs were co‐transfected with pRL‐SV40 to standardize the transfection efficiency. Data presented are the mean ± SD. Each cell line was used in 3 independent transfection experiments, and each experiment was performed in quadruplicate.*** represents *P* < 0.001

**Figure 3:**
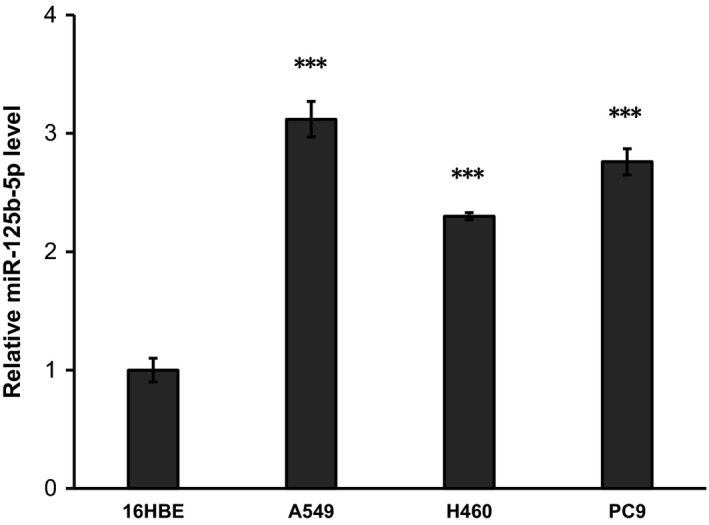
Significantly higher relative miR‐125b‐5p level in lung cancer cell lines (A549, H460, PC9) compared with normal human bronchial epithelial cell line (16HBE). All reactions were performed in triplicate and are presented as mean ± SD. ***P < 0.001

According to the SNPinfo, rs2241490 and rs512932 might modulate the binding of transcription factor. Thus, we hypothesized rs2241490‐A and rs512932‐G might influence the hsa‐miR‐125b‐1 expression. We generated four luciferase reporter gene plasmids (rs2241490 G and A allele; rs512932 A and G allele) and used pRL‐SV40 plasmids to normalize the transfections. Significantly higher levels of luciferase activity were observed for the reporter gene vector with rs512932 G allele than that with A allele in 293T, SPC‐A1 and A549 cells (7.810 vs 1.009, *P* = 0.002; 9.119 vs 0.831, *P* = 0.002; 8.206 vs 0.691, *P* < 0.001, respectively, Figure 2). However, no significantly different levels of luciferase activity were observed when we transfected luciferase reporter gene plasmids carrying the rs2241490‐G allele or carrying the rs2241490‐A (293T: 0.939 vs 0.756, *P* = 0.140; SPCA1: 0.980 vs 0.826, *P* = 0.100; A549: 0.946 vs 0.866, *P* = 0.250, respectively, Figure 2). These results suggested that rs512932 A > G might upregulate the expression of miR‐125b‐1 by increasing transcriptive activity. In addition, according to the real‐time PCR assay, miRNA‐125b had significantly higher expression in lung cancer cells when compared with 16HBE cells (*P* < 0.001) (Figure 3).

## 4 DISCUSSION

In this study, we focused on six potentially functional SNPs in miR‐125 family (miR‐125a, miR‐125b‐1, miR‐125b‐2) and found that rs2241490, rs512932 and rs8111742 were associated with the prognosis of NSCLC patients in a Chinese population, and the combined analysis of the three SNPs showed a significant locus‐dosage effect of the number of risk alleles (rs2241490‐A, rs512932‐G and rs8111742‐G) on NSCLC survival. Furthermore, luciferase reporter gene assay showed that rs512932‐G could increase the transcription activity of miR‐125b‐1. To the best of our knowledge, this is the first clinical follow‐up study to evaluate the association between germline genetic variants in miR‐125 family and NSCLC survival in Chinese population.

Many studies have highlighted the role that germline variants in miRNAs or regulatory elements played in cancer.^18^, ^22^ In this study, we found that rs2241490 (228bp upstream of pre‐miR‐125b‐1), rs512932 (2989bp upstream of pre‐miR‐125b‐1) and rs8111742 (1033bp upstream of pre‐miR‐125a) were significantly associated with the survival of NSCLC patients, and the G allele of rs512932 increased the transcriptional activity. According to HaploReg,^23^ rs512932 in A549 is marked with enhancer activity (H3K4me1^24^and chromatin states 25‐state model), in normal human lung fibroblasts (NHLF) is marked as both enhancer (H3K4me1, H3K27ac) and promoter (H3K4me3) and also as 5’ preferentially transcribed. The regulatory motifs possibly altered by the SNP are CDP.^25^ In addition, it showed that rs512932 is an eQTL of lnc‐RNA RP11‐166D19.1 (ENSG00000255248.2, slope=0.085, *P* = 0.013) according to GTExV7.^26^ RP11‐166D19.1 is an isoform of MIR100HG, which is a leukemia‐related oncogene^27^ hosting three miRNAs (*let‐7a*, *miR‐100*, and *miR‐125b‐1*) as a cluster in its introns.^28^ We did not find any evidence for rs512932 regarding eQTLs toward hsa‐mir‐125b‐1/hsa‐miR‐125b‐5p/hsa‐miR‐125b‐1‐3p in GTEx or TCGA lung adenocarcinoma (LUAD)/squamous cell lung carcinoma (LUSC) samples. However, there might still be a possible mechanism in different cell context that rs512932 modify the transcription of miR‐125b‐1 with further functional studies to reveal the relationships among the SNP, miR‐125b‐1, MIR100HG and RP11‐166D19.1.

miR‐125b is transcribed from two loci located on chromosome 11q24 and 21q21 with corresponding product of hsa‐miR‐125b‐1 and hsa‐miR‐125b‐2,^16^ respectively. Studies have shown that miR‐125b, as an oncogene, is highly expressed in lung cancer cells/tissues/serums and is associated with poor outcome of NSCLC patients.^29^, ^30^, ^31^, ^32^, ^33^ Our study indicated that the G allele of rs512932 might play a role in the development and prognosis of non‐small‐cell lung cancer through affecting the binding of the transcription factor, which then alters miR‐125b expression. Rs2241490 was not associated with the transcriptional activity of miR‐125b‐1. As it was predicted by chromatin states 25‐state model that rs2241490 in A549 has promoter activity, the SNP may be associated with NSCLC survival through other mechanisms. Further function‐based studies are warranted to verify our findings.

Most of the studies on miR‐125a‐3p/5p found that it was a tumor suppressor and was downregulated in lung cancer. Higher expression of miR‐125a may predict better survival in NSCLC patients.^14^, ^34^, ^35^, ^36^, ^37^Wang *et al* reported that miR‐125a, as a metastatic suppressor in lung cancer cells, activated by epidermal growth factor receptor (*EGFR*) signaling, inhibits tumorigenesis and tube formation.^38^ In our study, rs8111742 located 1033bp upstream of miR‐125a was associated with better survival in NSCLC patients. The SNP in A549 is marked by both enhancer (H3K4me1 and H3K27ac) and promoter (H3K4me3 and H3K9ac) according to HaploReg, indicating the region is active regulatory elements. It is possible that rs811742 might change the activity of the regulatory elements that harbor it, thus change the expression of miR‐125a, which is associated with the survival of NSCLC.

Several limitations of our study are needed to be addressed. First of all, a relatively small sample size could confine the statistical power of the study, especially in the interaction analysis, and additional larger scale population‐based studies are needed to reinforce the reliability of our results. Secondly, as a hospital‐based study, intrinsic selection bias cannot be entirely excluded. Thirdly, when taking multi comparison into consideration, two SNPs remained significant (*P*_adj_=0.023 for both rs2241490 and rs512932) except rs8111742 (*P*_adj_ = 0.056) in dominant models after using false discovery rate (FDR). Finally, although higher luciferase activity of reporter plasmids containing rs512932 variant G allele in three cell lines was observed, evidence from lung cancer tissue with the same origin of the blood specimen analyzed was limited. And we were unable to clarify real biological effects derived from allele difference. Further functional studies on cell lines or tissues may help to confirm and expand our findings. Nevertheless, this is the first ever to examine the association between the polymorphisms of miR‐125 family and prognosis of NSCLC, and provided valuable information for future researches and clinical practice.

This study indicated that rs2241490, rs512932 and rs8111742 in miR‐125 family were associated with the prognosis of NSCLC patients in a Chinese population. Larger population‐based and functional studies are needed to verify these findings.

## CONFLICT OF INTEREST

The authors have declared that no competing interests exist.
